# Lovastatin and Resveratrol Synergistically Improve Wound Healing and Inhibit Bacterial Growth

**DOI:** 10.3390/ijms26020851

**Published:** 2025-01-20

**Authors:** Norah A. AlJunaydil, Rhodanne Nicole A. Lambarte, Terrence S. Sumague, Osama G. Alghamdi, Abdurahman A. Niazy

**Affiliations:** 1 Department of Oral and Maxillofacial Surgery, College of Dentistry, King Saud University, Riyadh 11451, Saudi Arabia; norahaljunaydil@gmail.com (N.A.A.); oghamdi@ksu.edu.sa (O.G.A.); 2 Molecular and Cell Biology Laboratory, Prince Naif bin Abdulaziz Health Research Center, College of Dentistry, King Saud University Medical City, King Saud University, Riyadh 11545, Saudi Arabia; rlambarte@ksu.edu.sa (R.N.A.L.); tsumague@ksu.edu.sa (T.S.S.); 3 Department of Oral Medicine and Diagnostic Sciences, College of Dentistry, King Saud University, Riyadh 11545, Saudi Arabia

**Keywords:** lovastatin, resveratrol, cell viability, wound healing, *P. aeruginosa*, *S. aureus*, antibacterial, synergistic effect, cell proliferation, scarring

## Abstract

Wound healing is a complex physiological process, with scarring and infection caused by *Staphylococcus aureus* and *Pseudomonas aeruginosa* being the most common complications. The reutilization of known medications has received increased attention for their role in cell function as small molecules. Examples of these include lovastatin, a cholesterol-lowering agent, and resveratrol, which have multiple biological properties. Both molecules have been reported to improve wound healing and possess antibacterial properties, with conflicting results. The wound-healing capabilities of human mesenchymal stem cells were evaluated after exposure to lovastatin, resveratrol, and their combination through scratch test, migrations assay, and qPCR. Protein docking was performed to assess the lovastatin/resveratrol combination as potential wound-healing targets. AlamarBlue assay was used to determine cell viability. Additionally, the impact of lovastatin and resveratrol combination to inhibit the growth of *S. aureus* and *P. aeruginosa* was tested using broth microdilution test and checkerboard assay to determine synergism. The combination of lovastatin 0.1 μM and resveratrol 0.1 μM synergistically improved wound healing and demonstrated an additive effect against *S. aureus* and *P. aeruginosa*, presenting potential antibacterial applications.

## 1. Introduction

Wound healing is a complex and distinctive cascade of overlapping events that regenerates or repairs dysfunctional tissues after an injury caused by accidents or trauma [1,2]. This cascade comprises three distinct phases: inflammatory, proliferative, and remodeling [3]. Any disturbance in the wound-healing dynamic results in complications and delayed wound healing, particularly in the inflammatory and proliferative stages [4,5]. Despite the body’s unique ability to heal, any disruption of the wound-healing cascade can result in significant morbidity to the patient and additional burden to the healthcare system, with surgical wounds costing the highest wound-related expenses, followed by diabetic foot injuries [6,7]. For instance, disruption of the inflammatory phase can result in wound infection, particularly around days 3–7 after injury [8]. The infection process occurs either due to the patient’s reduced immune response, an increase in microorganism biofilm, or a combination of both [9,10].

Infection of the wound can further damage the tissues, delay the healing process, and increase scarring [11]. *S. aureus* and *P. aeruginosa* are the most common bacterial cause of wound infections, and targeting these bacteria in wound hygiene would reduce the risk of infection [9,12,13].

Regenerative tissue engineering utilizes different biomaterials, including small molecules, to modulate stem cell function to regenerate impaired and injured tissues [14]. Mesenchymal stem cells (MSCs) are the primary cells used in various tissue engineering processes, including studies and treatments related to wounds, due to their low immunogenicity, relatively easy harvest, and availability in different tissues. In addition, the role of MSCs in tissue healing is not limited to their differentiation ability; rather, they play a crucial part in modulating the various stages of wound healing by managing the inflammatory phase and enhancing the proliferation and function of fibroblasts and myofibroblasts in the proliferative stage [15,16,17].

**Figure 1:**
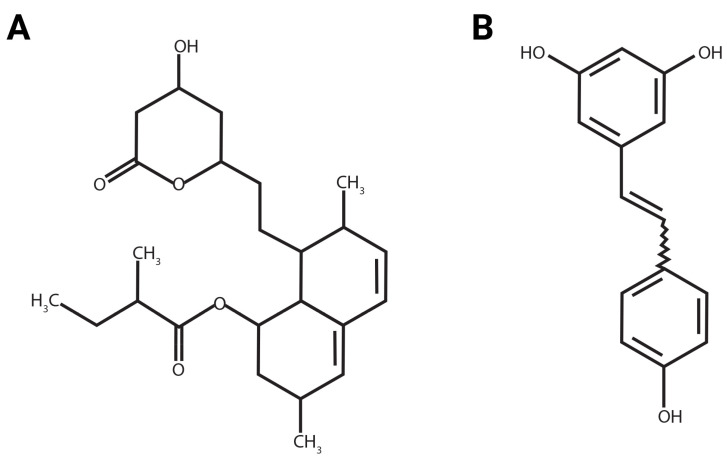
Chemical structure of (**A**) lovastatin and (**B**) resveratrol.

One of the commonly investigated small molecules is the statin family, which are widely used as cholesterol-lowering agents that function by inhibiting the function of 3-hydroxy-3-methylglutaryl coenzyme A (HMG-CoA) reductase [18,19]. Lovastatin (Lov), a secondary metabolite produced by the fungi *Monascus ruber* and *Aspergillus terreus*, was the first drug from the statin family to be approved by the Food and Drug Administration (FDA) (Figure 1A) [20].

As lovastatin became widely available in the market, this led to its increased use in clinical practice as well as expanding research on its use as a small molecule [18,21,22]. Lovastatin was shown to have a polyfunctional effect on different levels of wound healing and carried antibacterial properties in addition to immunomodulation and anti-inflammatory function [23].

Another example of a commonly used small molecule is a polyphenol known as resveratrol (3,5,4′-trihydroxy-trans-stilbene) (Figure 1B). This small molecule is naturally found in more than 70 species of plants, including grape skin and seeds, as well as in infected plants, especially those affected by fungi or ionizing radiation [24,25]. Resveratrol (0.1–100 μM, Res) has multiple biological properties as a cardioprotective, anticancer, antioxidant, neuroprotective, and anti-inflammatory agent [24,25]. Furthermore, resveratrol promoted wound healing as it increased cell migration to the injured area, had an anti-inflammatory function, and helped in controlling collagen production [13,26].

Several studies have reported the effectiveness of combining statins and resveratrol. However, the systematic administration of resveratrol and lovastatin has been questioned owing to their poor water solubility and rapid metabolism, which may affect their bioavailability. This has opened the door for further exploration of local delivery systems [27,28,29,30,31].

However, local delivery presents its own challenges, such as determining the safe concentration of a specific drug for direct application to cells. Additionally, the possible interactions of these drugs could alter their safety levels for local delivery. In in vitro studies, the cytotoxic levels of lovastatin varied significantly based on the cell line and the duration of exposure. Some studies have reported 10 μM lovastatin-induced apoptosis in lung cancer cells after 72 h of exposure, whereas 20 μM lovastatin was cytotoxic to ovarian cancer cells in only 24 h [32,33]. In addition, concentrations of 8 μM and higher were toxic to pluripotent mesenchymal cells despite the duration of exposure [34].

As with lovastatin, the cytotoxic effect of resveratrol is influenced by the duration of exposure and the type of cells. For instance, high doses of resveratrol (10–100 μM) induce cell death in colon cancer cells [35], whereas only resveratrol 15 μM has a cytotoxic effect on bone mesenchymal stem cells (BMSCs) [36].

In addition, the repurposing of existing small-molecule drugs as antibacterial agents has increased due to the emergence of antibiotic-resistant bacteria. Various bacterial species, including Gram-positive and Gram-negative strains exposed to lovastatin and resveratrol, yielded varying results [37,38]. Both lovastatin and resveratrol showed promising results for Gram-positive bacteria, particularly the antibacterial effect of lovastatin against some drug-resistant pathogens [39]. However, only resveratrol demonstrated growth inhibition in Gram-negative species [40].

Furthermore, drug repurposing using molecular docking is one of the major in silico tools for the drug-discovery approach, which reduces drug development and cost [41]. Docking studies provide preliminary valuable insights into the molecular structure in determining the mechanism of action and synergistic activity and further investigate the potential stable affinity of the compound towards the target molecule [42]. AutoDock Vina software (version 1.1.2) is a commonly used docking software known for its precise and effective ligand and receptor interaction prediction [43].

This study investigated the effects of lovastatin, resveratrol, and their combination on the viability of human mesenchymal stem cells and their impact on wound healing. Furthermore, the antibacterial and synergistic effects of lovastatin and resveratrol against Gram-positive *S. aureus* and Gram-negative *P. aeruginosa* were tested.

## 2. Results

### 2.1. Cell Viability for Individual Drugs

**Figure 2:**
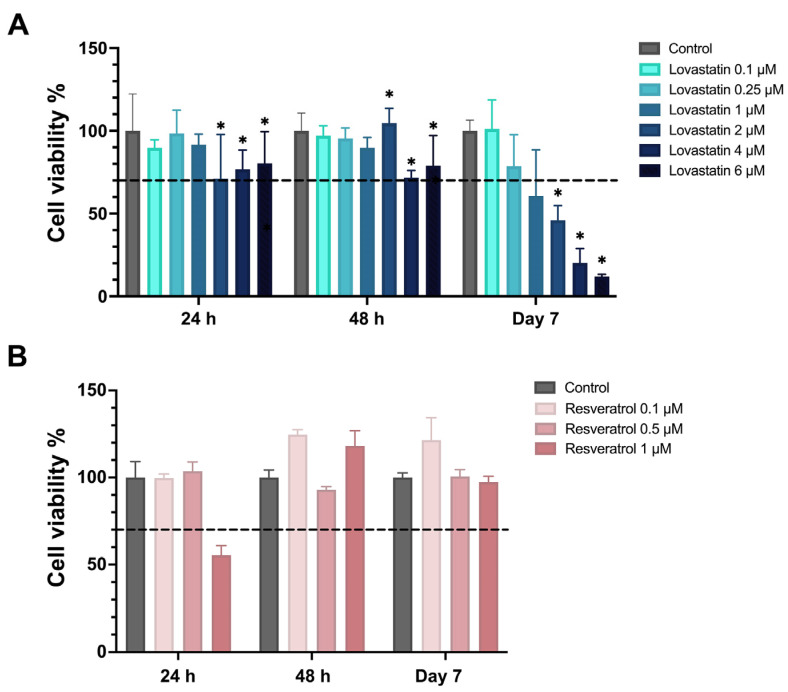
Cell viability percentages for TERT-20 hMSCs treated with (**A**) lovastatin and (**B**) resveratrol. Data presented as mean ± SD of cell viability percentage, tested using two-way ANOVA, Tukey’s test, (* *p* < 0.05 compared to the control group). The dotted line indicates 70% cell viability.

Following 24 and 48 h of exposure to lovastatin, the 0.1, 0.25, and 1 µM lovastatin groups had significantly higher viability percentages after 24, 48 h, and day 7 of exposure (Figure 2A). Furthermore, there was a significant reduction (*p* < 0.001) in cell viability in lovastatin at higher concentrations (4 and 6 µM) compared to the control group, which also extended to day 7. In addition, despite the recovery of cells after 48 h of exposure to 2 µM (*p* < 0.001), the cell viability was significantly below the 70% viability threshold at day 7 and was significantly lower than the control group (*p* < 0.01). Despite an increase in cell viability in the 0.1 μM resveratrol-exposed group compared to the control group (Figure 2B), resulting in 124.7% and 121.4% after 48 h and day 7, respectively (*p* > 0.05). However, reduced cell viability was below the 70% threshold at 24 h after exposure to 1 μM of resveratrol but showed a slight increase at 48 h and a comparable response to the control at 7 days.

### 2.2. Cell Viability for Lov/Res Combinations

**Figure 3:**
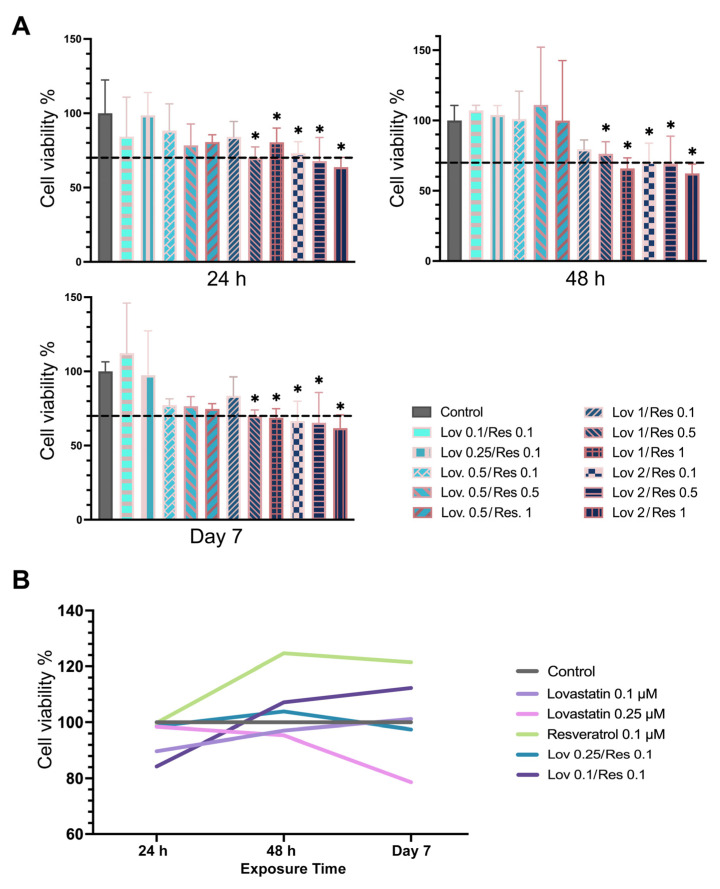
Cell viability percentage for (**A**) Lov/Res combinations at different time intervals and (**B**) linear trend of the least toxic concentration of Lov, Res, and Lov/Res. Data presented as mean ± SD of cell viability percentage, tested using two-way ANOVA, Tukey’s test (* *p* < 0.05 compared to the control group). The black dotted line indicates 70% cell viability.

Based on the findings of cell viability after exposing hMSCs-TERT 20 to lovastatin and resveratrol, Lov 2/Res 0.1, Lov 2/Res 0.5, Lov 2/Res 1, Lov 1/Res 0.1, Lov 1/Res 0.5, Lov 1/Res 1, Lov 0.5/Res 0.1, Lov 0.5/Res 0.5, Lov 0.5/Res 1, Lov 0.25/Res 0.1, and Lov 0.1/Res 0.1 were tested. Despite the different concentrations of resveratrol, the lovastatin concentration in the combinations significantly influenced cell viability compared to the control group over time, which is evident in lovastatin 1 and 2 μM (*p* < 0.01) (Figure 3A). The lovastatin 1 μM/resveratrol 0.1 μM combination had no significant reduction compared to the control; however, the cell viability percentage was below 70% by day 7 (*p* > 0.05). Moreover, the lovastatin 0.1/resveratrol 0.1 combination increased viability to 112.3% on day 7 compared to the control (*p* > 0.05).

By comparing the highest cell viability percentage between the drugs and their combinations over time (Lov 0.1 μM, Lov 0.25 μM, Res 0.1 μM, Lov 0.1/Res 0.1, and Lov 0.25/Res 0.1), there is increased viability after exposing the cells to 0.1 μM of lovastatin on day 7 compared to 24 h and 48 h (*p* < 0.001). However, only resveratrol 0.1 μM had significantly increased cell viability compared to lovastatin 0.25 μM in 24 h and 48 h and day 7 (*p* < 0.01) (Figure 3B).

### 2.3. Cell Morphology

**Figure 4:**
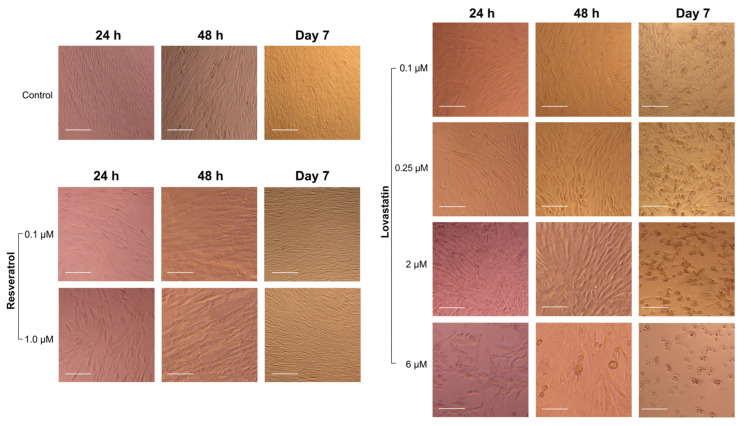
Cell morphology after exposure to different concentrations of lovastatin and resveratrol at 3-time intervals (5× magnification). Scale bar: 100 µm.

The TERT-20 hMSCs morphology was affected by the concentration and duration of the exposure to either lovastatin, resveratrol, or both. Early morphological changes were noted in the lovastatin group in doses >4 μM with evident cell detachment in 6 μM wells (Figure 4). After 48 h of exposure to lovastatin (≥2 μM), TERT-20 hMSCs became thinner and elongated, with some signs of detachment. However, by day 7 of exposure, the TERT-20 hMSCs cells were rounded and detached with an enlarged nucleus. In addition, by day 7 of exposure to lovastatin (≥0.25 μM), the cell morphology changed to spindled cells with an off-centered nucleus.

On the other hand, the TERT-20 hMSCs changed in morphology immediately after 24 h of exposure to Res 1 μM; the cells became thinner and spread out throughout the well but then started to show signs of recovery and returned to the sigmoid, flattened shape after 48 h and continued to recover until day 7 to become similar to the control group.

**Figure 5:**
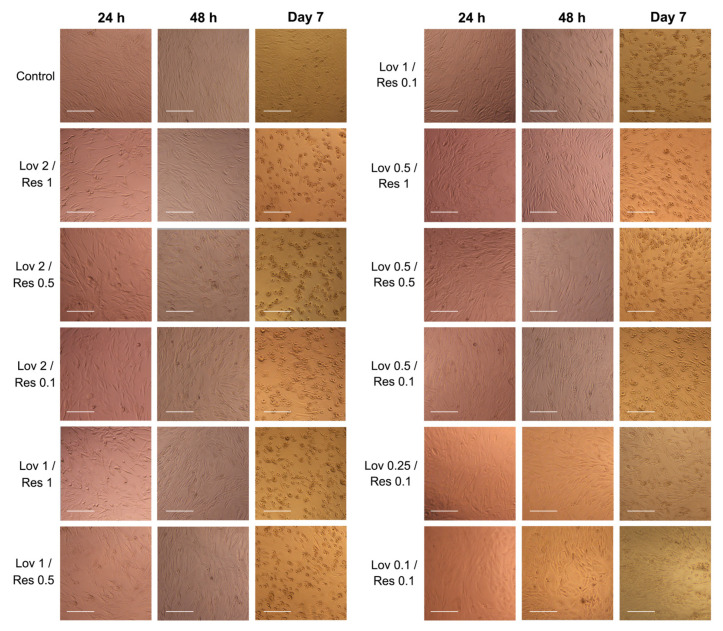
Cell morphology after exposure to different concentrations of lovastatin/resveratrol combinations at 3-time intervals (5× magnification). Scale bar: 100 µm.

In the combination experiment, the TERT-20 hMSCs morphology was greatly affected by the concentration of lovastatin despite resveratrol concentration (Figure 5), particularly in lovastatin concentration (≥2 μM), which can be noted as early as 24 h. In addition, the Lov 0.1/Res 0.1 and Lov 0.25/Res 0.1 combination exhibited the least significant impact on cell morphology throughout exposure, with the cells remaining sigmoid and flat.

### 2.4. Scratch Wound-Healing Assay

**Figure 6:**
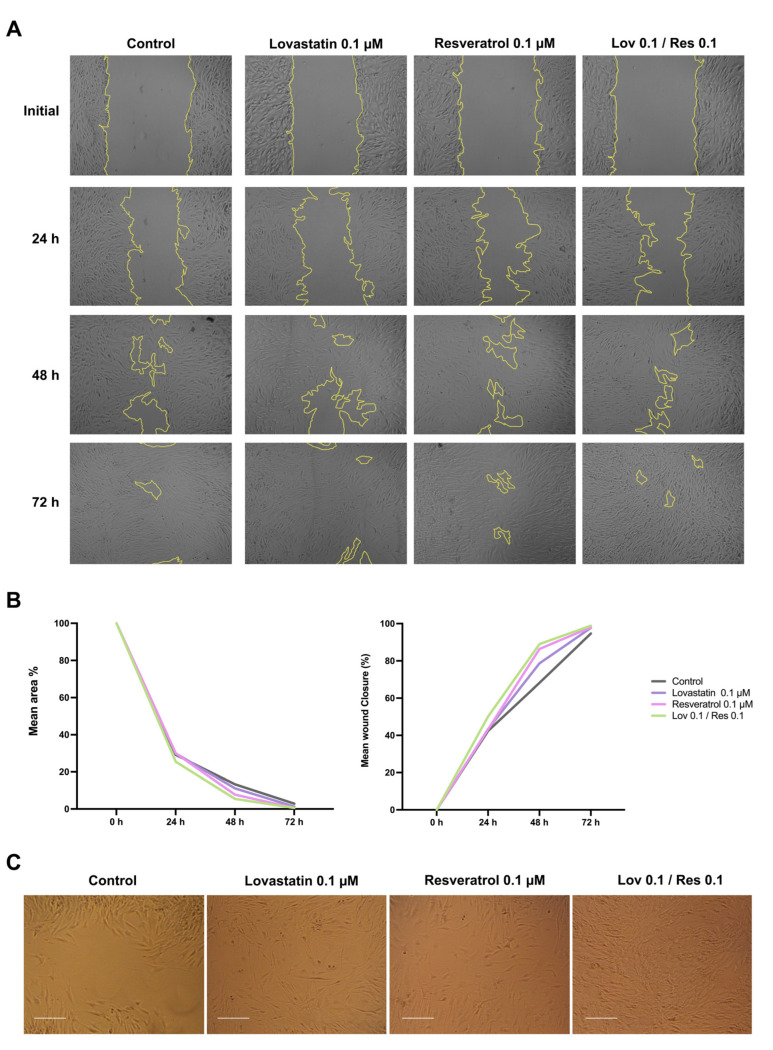
Scratch wound-healing assay: (**A**) quantification of wound closure over time, yellow lines indicate the scratch area, (**B**) mean area percentage and mean wound closure percentage over time, and (**C**) images of the scratch at 48 h using 5× magnification. Data were tested using two-way ANOVA, Tukey’s test (*p* < 0.05 compared to the control group). Scale bar: 100 µm.

Based on the findings of the cell viability study, Lov 0.1 μM, Res 0.1 μM, and Lov 0.1/Res 0.1 were used for further testing. Following quantifying the images obtained at 24, 48, and 72 h after inducing the scratch, the mean wound area and the rate of closure were significantly different between each time interval (*p* < 0.01) (Figure 6A).

Lov 0.1*/*Res 0.1 had the highest rate of wound closure in all time intervals. At 24 h, the wound was 50% reduced to its original size. However, lovastatin 0.1 μM and resveratrol 0.1 μM showed the same closure rate of 43%, which was close to the control rate of 42% (Figure 6B).

Furthermore, at 48 h, the wound area of the Lov 0.1/Res 0.1 and resveratrol 0.1 μM groups was reduced to 5% and 7.7%, respectively, compared to 13.3% in the control group.

In addition, the cell orientation significantly differed between the groups (Figure 6C). In Lov 0.1/Res 0.1, the cells resembled the adjacent cells in terms of elongation and direction, with very few perpendicular crossing cells. Furthermore, in Res 0.1 μM, the gap between the ends of the scratch is more evident with more irregularly shaped cells and condensation of cells on the periphery of the wound, while in Lov 0.1 μM had less cell condensation at the periphery but more irregular and the cells were oriented in a crosshatched pattern. However, in the control group, the cell condensation at the scratch edges was more evident, with obvious gaps and more perpendicular cells that had an irregular, short shape in the center and were more elongated at the wound edges.

By 72 h, the experiment groups had the highest wound closure rates: 99% in Lov 0.1/Res 0.1, 98% in lovastatin 0.1 μM, and 98% in resveratrol 0.1 μM with indistinguishable cells from non-scratched areas, particularly in Lov 0.1/Res 0.1, while the control group had only 95% closure and more perpendicular cells distinguishing the site of the scratch.

### 2.5. Transwell Migration Assay

**Figure 7:**
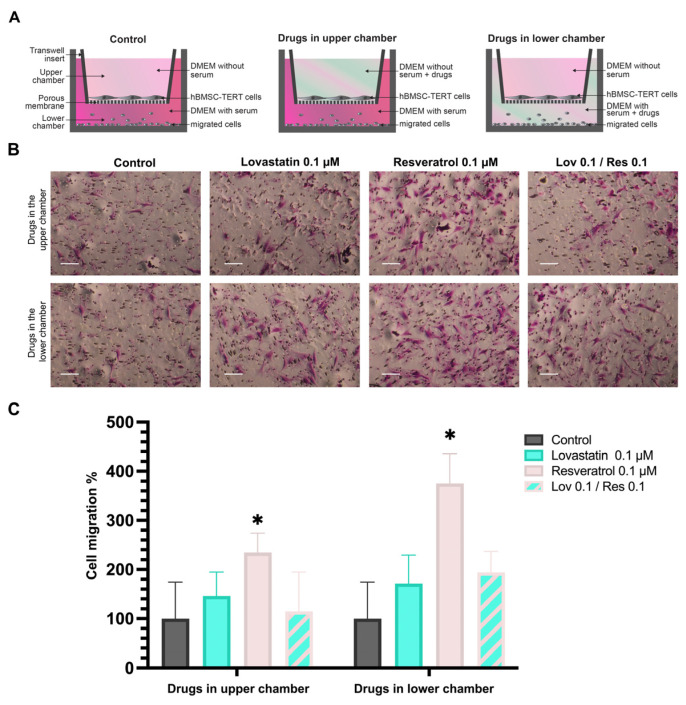
Transwell migration assay: (**A**) experiment protocols, (**B**) captured images of migrated cells (20× magnification, scale bar: 100 µm), and (**C**) mean ± SD cell migration percentage tested using two-way ANOVA, Tukey’s test, (* *p* < 0.05 compared to the control group).

Generally, after adding Lov 0.1 μM, Res 0.1 μM, and Lov 0.1*/*Res 0.1 in the lower chamber, the human TERT-20 MSCs migrated more compared to the control wells (Figure 7). In addition, when the same concentrations were added in the upper chamber compared to adding them in the lower chamber, the cells with Lov 0.1 μM, Res 0.1 μM, and Lov 0.1*/*Res 0.1 in the lower chamber showed more migration.

However, only Res 0.1 μM in the lower chamber showed a significant increase in cell migration compared to Res 0.1 μM in the upper chamber (*p* < 0.01). In addition, Res 0.1 μM wells had more migrated cells compared to the control (*p* < 0.1), with a mean percentage of 376%.

Furthermore, Lov 0.1*/*Res 0.1, in the lower chamber, migration mean was higher than those in the Lov 0.1 μM group, 194% and 171%, respectively. Meanwhile, Lov 0.1*/*Res 0.1 added to the upper chamber had a mean of 115% compared to 146% of Lov 0.1 μM in the upper chamber.

### 2.6. Real-Time Quantitative Polymerase Chain Reaction (qPCR) of Wound-Healing-Related Genes

**Figure 8:**
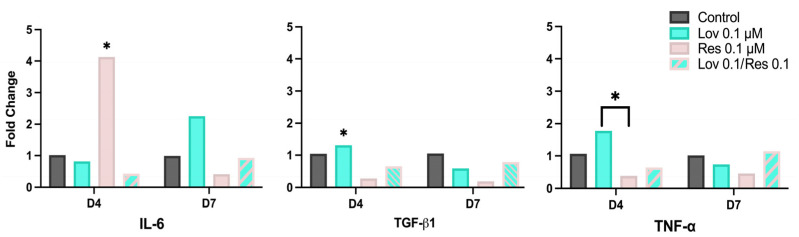
IL-6, TGF-β1, and TNF-α mRNA expression (fold change) at days 4 and 7 for treated-hMSCs with lovastatin, resveratrol, and their combination, tested using two-way ANOVA, Tukey’s test (* *p* < 0.05).

qPCR was conducted to identify the expression of wound-healing-related genes, including interleukin-6 (IL-6), transforming growth factor-β1 (TGF-β1), and tumor necrosis factor-α (TNF-α) (Figure 8).

At day 4, IL-6 was significantly high in Res 0.1 μM up to 4-fold when compared to Lov 0.1*/*Res 0.1 at day 4 (*p* < 0.05). However, Res 0.1 μM exposed cells reduced the IL-6 expression to 0.4-fold by day 7 (*p* < 0.05), which was the lowest among the groups.

In addition, IL-6 in the Lov 0.1 μM group increased from 0.8-fold on day 4 to 2.25-fold on day 7, while a minimal increase was noted in Lov 0.1*/*Res 0.1 with only a 0.5-fold difference between the two time intervals (0.4 on day 4 to 0.9 on day 7).

The overall expression of TGF-β1 was downregulated and generally reduced over time, except in the Lov 0.1*/*Res 0.1 group, which increased from 0.65 to 0.79-fold between days 4 and 7. However, only Lov 0.1 μM on day 7 was significantly lower than on day 4 (*p* < 0.01). In addition, resveratrol showed the least expression of TGF-β1, particularly on day 7 (0.2-fold).

On the other hand, only TNF-α expression was upregulated with Lov 0.1 μM at day 4 and was significantly increased compared to Res 0.1 μM (*p* < 0.05). While Lov 0.1*/*Res 0.1 doubled the expression of TNF-α by day 7 (0.6 to 1.4) and was the only group that was upregulated, Res 0.1 μM showed negligible change over time.

### 2.7. Combined Drugs Molecular Docking Studies

**Figure 9:**
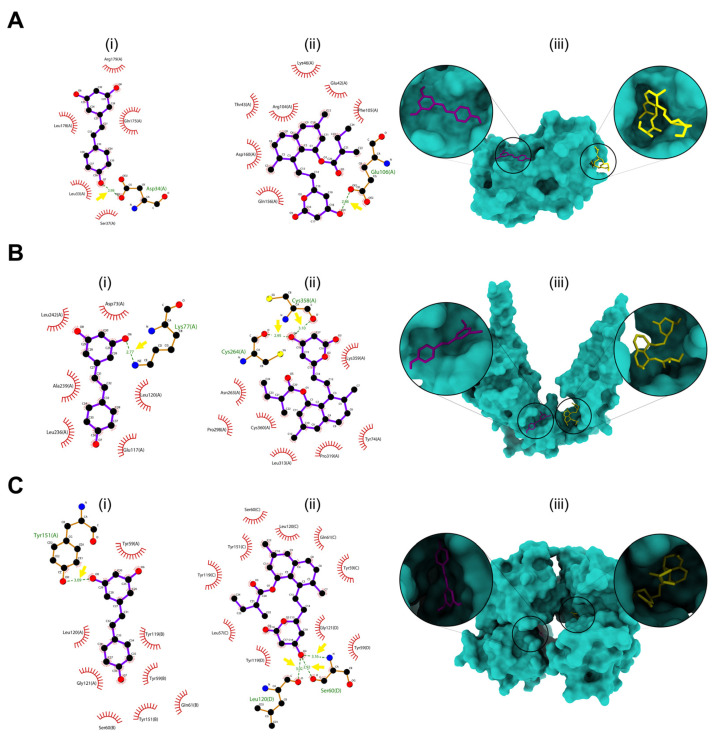
Molecular-docked complexes of (**A**) IL-6, (**B**) TGF-β1, and (**C**) TNF-α of combined ligands (i) Res and (ii) Lov. LigPlot interaction diagram of (i) Res, (ii) Lov, and (iii) 3D surface placement of combined ligands; Res is represented as purple, and Lov is represented as yellow within the binding site. The yellow arrow indicates a hydrogen bond.

Molecular docking studies were performed among different structures of IL-6, TGF-β1, and TNF-α with the Lov*/*Res drug combination (Figure 9A–C). This study was performed to identify the potential mechanism of combined drugs’ action on wound-healing transcriptional regulators. The highest binding affinities for each receptor are shown in Table 1, and the detailed results are outlined in Supplementary Tables S2 and S3. The drug combination docking simulation for predicted wound-healing genes showed −6.0, −5.5, and −6.5 for IL-6, TGF-β1, and TNF-α, respectively. TNF-α and Lov*/*Res combination showed the highest binding affinity with a docking score of −6.5 and four H-bonds residues compared with IL-6 and TGF-β1 Lov/Res combination. On the other hand, IL-6 and Lov*/*Res combination showed a relative docking score with TNF-α and Lov*/*Res combination.

### 2.8. S. Aureus Growth Inhibition

**Figure 10:**
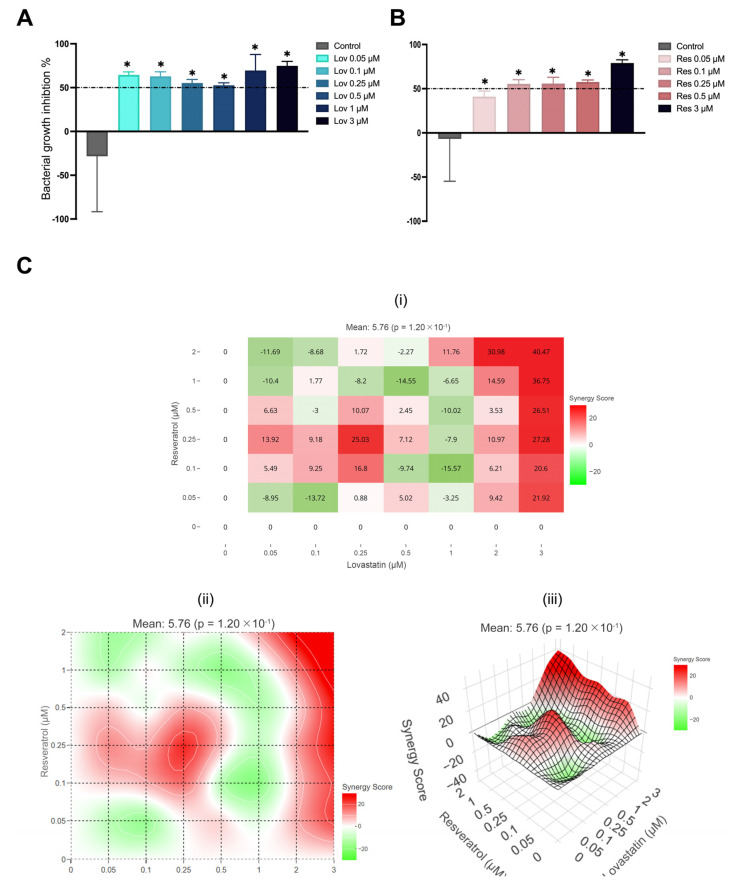
*S. aureus* growth inhibition: (**A**) after exposure to lovastatin, (**B**) after exposure to resveratrol, the data are presented as bacterial growth inhibition percentage tested using one-way ANOVA and Brown-Forsythe test with the threshold bar set at 50%, and (**C**) checkerboard result, data presented as synergy score (i) inhibition percentage dose-response matrix, (ii) 2D heatmap synergy scoring and (iii) 3D interaction landscapes (* *p* < 0.05 compared to the control group).

Despite the significant inhibition of bacterial growth after exposure to different doses of lovastatin (*p* < 0.01) (Figure 10A), the percentage of growth inhibition decreased as the dose of lovastatin increased (Lov 0.05 μM: 64.6%, Lov 0.1 μM: 62.8%, Lov 0.25 μM: 55.3% and Lov 0.5 μM: 52.3%). However, Lov 3 μM inhibited *S. aureus* growth by up to 75%.

On the other hand, as the dose of resveratrol increased, the percentage of inhibition increased slightly compared to the control (*p* < 0.01) (Figure 10B). In addition, all groups’ growth inhibition was above 50% of *S. aureus* growth except for Res 0.05 μM had only 41% inhibition, where Res 0.1 μM, Res 0.25 μM, and Res 0.50 μM had growth inhibition by 55%, 56%, and 58%, respectively. Conversely, as resveratrol doses increased to 3 μM, the percentage of *S. aureus* inhibition was 79%.

The SynergyFinder Plus package version 3.10.3 [44] was used to calculate each drug’s half-maximal inhibitory concentration (IC50): 1.51 μM for lovastatin and 0.33 μM for resveratrol. The impact of combining lovastatin and resveratrol on the growth of *S. aureus* was analyzed using Loewe’s synergy score, wherein the Lov 0.1/Res 0.1 combination showed an additive effect with a synergy score of 9.25 (Figure 10C); moreover, a synergistic effect was observed in the Lov 0.25/Res 0.1 and Lov 0.25/Res 0.25 combinations, with synergy scores of 16.8 and 25.03, respectively. However, the mean synergy score on *S. aureus* growth inhibition was insignificant (mean 5.67, *p* > 0.05).

### 2.9. P. Aeruginosa Growth Inhibition

**Figure 11:**
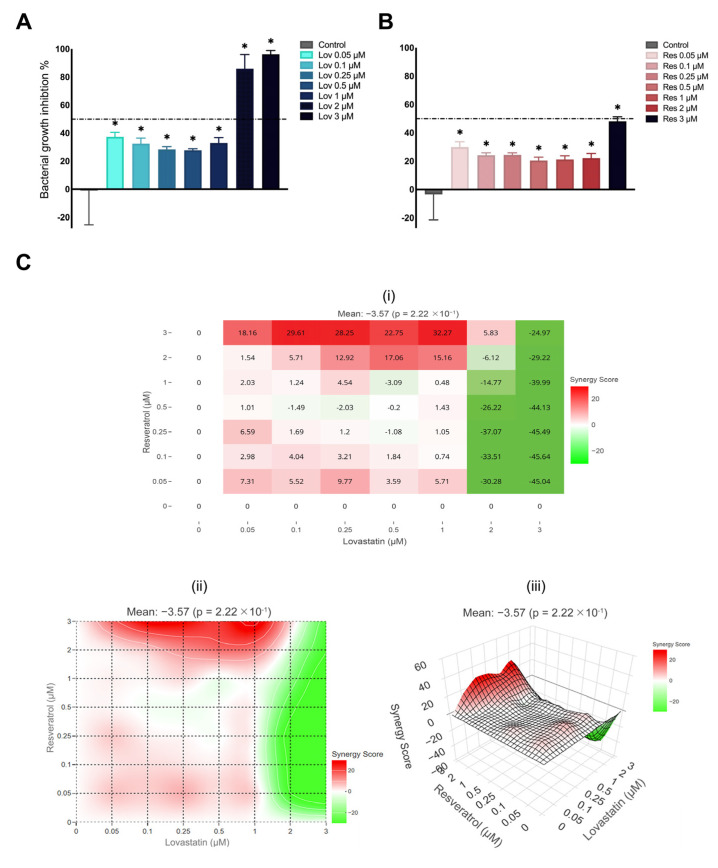
*P. aeruginosa* growth inhibition: (**A**) after exposure to lovastatin, (**B**) after exposure to resveratrol. The data are presented as bacterial growth inhibition percentage tested using one-way ANOVA and Brown-Forsythe test with the threshold bar set at 50%, and (**C**) checkerboard result, data presented as synergy score (i) inhibition percentage dose-response matrix, (ii) 2D heatmap synergy scoring and (iii) 3D interaction landscapes (* *p* < 0.5 compared to the control group).

Despite the significant reduction of *P. aeruginosa* growth after the exposure to lovastatin compared to the control (*p* < 0.01) (Figure 11A), the growth reduced by >85% only after exposure to 2 and 3 μM (86% and 96%, respectively). However, at lower concentrations of lovastatin (0.05, 0.1, 0.25, 0.5, and 1 μM), *P. aeruginosa* growth inhibition was reduced between 28 and 38%.

In addition, there was a significant reduction in *P. aeruginosa* growth after exposure to resveratrol (*p* < 0.01) (Figure 11B), yet the reduction was below the threshold of 50%. The percentage of growth inhibition ranged from 20 to 29% in 0.05–2 μM, and only 3 μM resveratrol reduced *P. aeruginosa* growth up to 48%.

Using the SynergyFinder Plus package version 3.10.3 [44], the IC50 was calculated for lovastatin as 3 μM, while resveratrol was 2.28 μM. In addition, the synergistic effect of lovastatin and resveratrol on *P. aeruginosa* was calculated (Figure 11C). The highest synergistic area score was 32.3, indicating a synergistic effect with the Lov 1*/*Res 3 combination. In addition, Lov 0.25*/*Res 0.1 and Lov 0.1*/*Res 0.1 combinations had a weak additive effect with scores of 3.21 and 4.1, respectively, with growth inhibition percentages below 50% for both combinations. Furthermore, Loewe’s synergy equation detected no significant synergistic effect with a mean of −3.6 (*p* > 0.05).

Nonetheless, all combinations of Lov 2 and 3 μM showed an antagonistic effect, particularly in Lov 3/Res 0.25, with Loewe’s score of −45.5 and an inhibition percentage of 55%.

## 3. Discussion

Interest in exploring FDA-approved medications or small molecules for other treatment strategies is growing, particularly since these compounds can interact and modify cellular function without activating the immune system or affecting structural integrity [22,45,46,47,48].

The response of human bone marrow-derived mesenchymal stem cells to different concentrations of lovastatin, resveratrol, and their combination was evaluated to determine their impact on cell viability, wound-healing potential, and bacterial growth inhibition.

At high doses of lovastatin (>4 μM), cell morphology of TERT-20 hMSCs was significantly affected after only 24 h of exposure, with a significant reduction in viability and detachment of the cells. However, at lower concentrations, the proliferation of TERT-20 hMSCs increased, with cell morphology maintained, particularly at Lov 0.1 μM, even after day 7 of exposure. This indicates that the concentration of lovastatin and duration of exposure impacted TERT-20 hMSCs response. Interestingly, these findings contradict the literature, wherein higher concentrations of lovastatin were considered non-cytotoxic to the cell lines investigated [32,33,34,49,50,51]. For example, in ovarian cancer cells, lovastatin 20 μM for 24 h induced cell apoptosis by blocking HMG-CoA reductase activity, whereas cytotoxicity was observed in lung cancer cells after exposure to Lov 10 μM for 72 h by increasing p21^WAF^ and/or p27^KIP^, and decreasing cyclin D1 which are cell-cycle checkpoint regulators [32,33]. These also indicate that the cell lines used in studies influence the cytotoxic dose of lovastatin. This also can be attributed to the mechanism of lovastatin to inhibit HMG-CoA reductase, which influences cell DNA synthesis, gene transcription, protein phosphorylation, and protein degradation [52].

Resveratrol at lower concentrations (particularly 0.1 μM) increased the proliferation of TERT-20 hMSCs and maintained their morphology. The highest cell viability was observed after 48 h of exposure; however, a slight decrease in viability was noted on day 7, which could be due to the over-confluence of cells in the wells. A similar response was noted in human mesenchymal stem cells, wherein a high concentration of resveratrol (≥5 μM) prolonged the cell doubling time, wherein Res 0.1 μM increased cell self-renewal [53]. In addition, resveratrol induced cell-cycle arrest through a p53-independent pathway in lung cancer cells (>25 µM), in addition to increasing pro-apoptotic factors like Bax to activate the intrinsic apoptotic pathway [54,55].

It was further observed that high concentrations (≥1 μM) of lovastatin in Lov/Res combinations negatively affected TERT-20 hMSCs cell viability and morphology as early as 24 h, similar to lovastatin treatment alone, which can be explained by the role of lovastatin in cell-cycle arrest at G_2_ phase by directly influencing the cholesterol synthesis through HMG-CoA reductase inhibition [52]. However, Lov 0.1/Res 0.1 progressively improved TERT-20 hMSCs proliferation and maintained cell morphology. Although the exact mechanism of promoting cell proliferation is still unknown, this can be attributed to statins’ ability to act on PI-3 K/Akt and AMPK antiapoptotic pathways; in addition, resveratrol’s ability to regulate the expression of the antiapoptotic *Bcl-2* gene [56,57].

The Lov 0.1, Res 0.1, and Lov 0.1/Res 0.1 μM groups were of interest for further investigation due to increased cell proliferation. The Lov 0.1 μM-induced group exhibited a network or crosshatched orientation pattern with reduced condensation at the edges and a faster wound-healing rate compared to the control, suggesting an increase in cell migration and a potential risk of scarring [58]. These findings also correlate with the migration pattern observed in the migration assay, wherein more cells migrated to the lower chamber upon the addition of lovastatin. This can be explained by the chemotactic ability of statins to attract cells, known as directional migration, favoring statins over regular cell media through the activation of the Akt/mTOR pathway, which may influence cellular migration and collagen production [59,60].

At day 4, Lov 0.1 μM increased both TNF-α and TGF-β1 expression, which could enhance cell migration and adhesion and promote inflammation during the early stages of wound healing. However, these markers also prolong the inflammatory phase and subsequently delay healing [61,62]. At day 7, Lov 0.1 μM increased IL-6 expression, which has been linked to scar tissue development, particularly in the late stages of wound healing, which was also observed in the scratch assay [63].

Moreover, Res 0.1 μM not only increased cell proliferation rate but also increased cell migration and wound healing, specifically when placed in the lower chamber, indicating its chemotaxis ability, possibly by enhancing Mn-SOD expression [59,64]. Res 0.1 μM also influenced the cell density at the wound edges and organized the cells in the bed, which can minimize the scarring as IL-6 significantly reduced at day 7, resulting in expected less scarring, which can be correlated with the upregulation of Mn-SOD expression [63,65]. TGF-β1 was also downregulated by Res 0.1 on both days 4 and 7, reducing it to 0.3 and 0.2-fold, respectively. This was linked to alterations in the TGF-β1/Smads signaling pathway, which plays a role in the reduction of pathological scarring [66].

Human TERT-20 MSCs exposed to Lov 0.1/Res 0.1 combination showed faster wound gap closure (up to 99%) and a more organized cellular layer compared to each drug used separately. Although the percentage of migration toward the lower chamber was midway between Lov 0.1 µM and Res 0.1 µM alone, the migration percentage was almost double that of the control group. The expressions of IL-6, TGF-β1, and TNF-α were notably reduced; however, it was insignificant. Overall, these data indicate an increased rate of wound healing and improved cellular structure at the wound site, enhancing the migration rate compared to the control.

Moreover, the molecular docking process was conducted using the crystal structures of IL-6, TGF-β1, and TNF-α [67,68,69]. These crystal structures were used to predict the binding affinity and interaction between Lov/Res combinations and the active sites of the enzyme. Thus, to ensure the conformational stability of the protein structure and the targeted interactions, an optimal state of hydrogen bonding energetics and kinetics is required [70]. The results obtained from molecular docking simulations provided insight into the potential mechanism of action of lovastatin and resveratrol in combination for the transcriptional regulators of wound healing.

In addition, lovastatin exerted a direct impact on the growth of *S. aureus* and *P. aeruginosa*, which are known to be the most common pathogens in wound infection [9,12,13]. At lower doses of lovastatin, had a higher *S. aureus* growth inhibition percentage as an initial response, whereas the inhibition percentage increased to 75% with Lov 3 μM. Therefore, the half-maximal inhibitory concentration (IC50) was calculated to be 1.51. As lovastatin acts on HMG-CoA reductase by inhibiting its function, *S. aureus* growth inhibition can be explained by the role of HMG-CoA reductase in isoprenoid biosynthesis [37,39].

Furthermore, the minimal inhibitory concentration (MIC95) of *P. aeruginosa* was Lov 3 μM, which correlated with the calculated IC50. The response of *P. aeruginosa* was significantly lower than those reported in the literature, as the MIC95 was often reported at much higher doses than those used safely for clinical practice [39,71]. The exact mechanism by which *P. aeruginosa* growth is inhibited is still unknown, given that it lacks HMG-CoA reductase protein [39,71]. Contrary to the promoted effect of resveratrol as an antibacterial compound [24,25], the percentage of *S. aureus* and *P. aeruginosa* growth inhibition was lower than that of lovastatin, yet the IC50 was lower.

In general, the percentage growth inhibition increased as the dose of resveratrol increased in both *S. aureus* and *P. aeruginosa*, wherein *S. aureus* percentage growth inhibition was more significant than those of *P. aeruginosa* with Res 3 μM reaching up to 79% inhibition. However, the IC50 [44] was 0.33 for *S. aureus* and IC50 2.28 for *P. aeruginosa*, respectively.

The MIC determined for *S. aureus* is >512 μg/mL, which is directly related to the number and location of hydroxyl groups in stilbenes [72]. On the other hand, *P. aeruginosa* MIC95 was 500 μg/mL, which was attributed to the outer membrane’s effective control of passing the amphipathic compounds and multidrug resistance pumps; hence, by blocking multidrug resistance pumps, the antibacterial activity of resveratrol against *P. aeruginosa* was improved [73].

Furthermore, the Lov 0.1/Res 0.1 combination showed an additive effect against both *S. aureus* and *P. aeruginosa*. Additionally, a synergistic effect was observed at a higher concentration (Lov 3/Res 2) against *S. aureus*, where the HMG-CoA reductase activity of lovastatin may have played a significant role by inhibiting HMG-CoA reductase and improving the antibacterial effect of resveratrol [71,72].

In contrast, Lov 3/Res 2 exhibited an antagonistic effect against *P. aeruginosa*; however, a synergistic effect was still noted as the resveratrol dose increased if the lovastatin dose was below 1 μM. Although the exact mechanism of lovastatin treatment against *P. aeruginosa* is still unknown, it may have a dose-dependent effect by promoting the penetration of resveratrol and improving its antibacterial properties [71,73].

One of the limitations of this study is that the cell line used for testing cell viability, TERT-20 hMSCs, is a telomerase-immortalized cell line that may influence cell behavior. In addition, the scope of the study was limited to exploring the potential of lovastatin, resveratrol, and their combination in a narrow aspect of wound healing. Third, the antibacterial effect of these small molecules was tested against only two strains of two bacterial species.

With these limitations, Lov 0.1/Res 0.1 showed an increase in cell proliferation and migration of human TERT-20 MSCs, in addition to faster wound healing with a monolayer that resembles the adjacent cells. Furthermore, Lov 0.1/Res 0.1 had an additive bacterial growth inhibition for both *S. aureus* and *P. aeruginosa*.

These results could warrant further exploration into the potential of local delivery of lovastatin-resveratrol combination directly to injured tissues and evaluate their effects in regenerative tissue engineering, particularly in cases involving specialized tissues such as bony defects. Furthermore, as bacterial wound infections are commonly observed in immunocompromised patients, such as those with diabetes, and the increasing prevalence of antibiotic-resistant bacteria, further investigation into the potential use of lovastatin and resveratrol as antibacterial agents against other bacterial species or as enhancers to existing antibiotics may be warranted.

## 4. Materials and Methods

### 4.1. Cell Culture

Immortalized human bone marrow-derived mesenchymal stem cells (TERT-20 hMSCs) were used to test the biocompatibilities of lovastatin and resveratrol and their combined effects. The characterized cell line was through the kind donation of Professor Moustapha Kassem, from the University of Southern Denmark, Odense, Denmark [74,75].

The cells were cultured in Dulbecco’s Modified Eagle’s Medium (DMEM, Thermo Fisher Scientific Inc., Waltham, MA, USA) with 4 mM glutamine, 1 mM sodium pyruvate, and 4500 mg/L glucose with additional supplementation of 1% non-essential amino acids, 1% penicillin-streptomycin and 10% fetal bovine serum (FBS) (all Gibco, Invitrogen, Carlsbad, CA, USA) and incubated in a 5.5% CO_2_ incubator at 37°C [49,76,77].

### 4.2. Drugs Preparation

Lovastatin and resveratrol compounds (Selleckchem Inc., Houston, TX, USA) were dissolved in 4 mL dimethyl sulfoxide (DMSO, Sigma-Aldrich, St. Louis, MO, USA) and then further diluted with DMEM (Gibco, Thermo Fisher Scientific Inc., Waltham, MA, USA) to obtain the different test concentrations [34,36,49,53]. For preliminary experiments, lovastatin at 0.1, 0.25, 0.5, 1, 2, 4, and 6 μM, whereas resveratrol at concentrations of 0.1, 0.5, and 1 μM was used.

Moreover, after the initial analysis of cell viability and morphology, different combinations of the least toxic doses of both drugs were used for further testing as a combination dose.

### 4.3. Cell Viability

The effects of lovastatin, resveratrol, and their combined doses on cell viability were evaluated using the AlamarBlue (AB) assay (Thermo Fisher Scientific, Waltham, MA, USA) according to the manufacturer’s instructions.

Briefly, 0.1 × 10^6^ cells/mL seeded in 96-well flat-bottom culture plates (NEST Scientific, NJ, USA) at 70–80% confluence was exposed to different doses of lovastatin, resveratrol, and their combinations.

Furthermore, by 24, 48 h, and day 7 of exposure to the drugs, 10 μL of AB solution was added to each well and incubated for 2 h at 37 °C [78]. The Synergy™ HT Microplate Reader (BioTek^®^ Instruments, Winooski, VT, USA) was used to measure the fluorescence intensities at λex = 560 nm and λem = 590 nm. The cell viability will be calculated using the following equation [76]:

(1) $$Cell\;viability\;\left(\%\right)=\frac{Fluorescence\;of\;the\;sample\;well}{Average\;fluorescence\;of\;control\;wells}\;x\;100$$

### 4.4. Cell Morphology

The cellular morphology was further evaluated under a phase-contrast microscope (Carl Zeiss Axiovert 40C Imaging Microscope, Göttingen, Germany) using a 5× objective to check for any evident cell damage, changes in cell morphology, or detachment. The digital photographs were viewed using ImageJ software, version 1.50i (National Institutes of Health, Bethesda, MD, USA), wherein two researchers conducted the evaluation independently.

### 4.5. Scratch Wound-Healing Assay

A 12-well culture plate (Greiner Bio-One GmbH, Frickenhausen, Germany) was seeded with 5 × 10^4^ cells/mL TERT-20 MSCs and was incubated overnight in a 5.5% CO_2_ incubator at 37 °C to reach 70–80% confluence. The scratch wound was created using a p200 pipette tip (NEST Scientific, Woodbridge, NJ, USA). To ensure consistency in wound dimensions, the plate cover was modified to create a straight, standard reference point when comparing the wells and for image acquisition.

Furthermore, the cells were washed with phosphate-buffered saline (PBS, Gibco, Thermo Fisher Scientific Inc., Waltham, MA, USA) and exposed to lovastatin 0.1 μM, resveratrol 0.1 μM or Lov 0.1/Res 0.1 based on the cell viability test results. Using a phase-contrast microscope (Carl Zeiss Axiovert 40C Imaging Microscope, ZEISS, Jena, Germany) with a 5× objective, six images per well were captured at the initial time point and 24, 48, and 72 h after inducing the wound at the same reference point.

The wound closure rate and mean wound area were quantified using ImageJ software version 1.50i (National Institutes of Health, Bethesda, MD, USA) [78,79]. Each measurement was repeated in triplicates, and the average was used.

### 4.6. Transwell Migration Assay

The migration of TERT-20 hMSCs was assessed using 24-well transwell chamber plates with an 8 μm pore size (NEST Scientific), as previously described, with some modifications [80,81].

Two protocols were used (Figure 7A); in the first protocol, the upper chambers were seeded with 1 × 10^3^ cells/mL with serum-free medium and incubated for 1 h, after which the lower chamber was filled with DMEM supplemented with lovastatin 0.1 μM, resveratrol 0.1 μM or Lov 0.1/Res 0.1. For the second protocol, 1×10^3^ cells/mL were incubated in serum-free DMEM for 1 h, followed by adding the chosen concentration of Lov, Res, and Lov 0.1/Res 0.1 to the upper chamber. The lower chamber contained DMEM without any drug supplementation.

After 24 h incubation at 37 °C, the migrated cells were fixed using 3.7% paraformaldehyde for 5 min and stained with 1% crystal violet (all Sigma-Aldrich), followed by washing with PBS thrice to remove excess staining solution. The non-migrant cells were gently removed from the upper chamber using a sterilized cotton swab. The number of migrated cells was quantified from six random microscopic fields/inserts using 20× magnification with an inverted microscope. The assays were independently repeated at least two times.

### 4.7. Real-Time Quantitative Polymerase Chain Reaction (qPCR) of Wound-Healing-Related Markers

After 24 h incubation of 6-well culture plates (Greiner Bio-One GmbH, Frickenhausen, Germany) seeded with 1 × 10^6^ cells/mL, the cells were exposed to lovastatin 0.1 μM, resveratrol 0.1 μM, or Lov 0.1/Res 0.1 in addition to the control group. To ensure validity, the experiment was repeated in triplicates on two independent days.

After 4 and 7 days of exposure, the wells were washed with PBS twice, followed by cell pellet collection. The expressions of wound-healing markers, namely interleukin-6, transforming growth factor-β1, and tumor necrosis factor-α, were measured, and their primer sequences were provided (Macrogen, Rockville, MD, USA) (Table 2) [82,83,84]. The HiGene™ Total RNA Prep Kit (BioFACT Co. Ltd., Daejeon, Korea) was used for RNA extraction according to the manufacturer’s instructions, and the quality and concentration were quantified using the BioSpectrometer^®^ Basic (Eppendorf, Germany).

This was followed by synthesizing complementary DNA (cDNA) by reverse transcription of RNA using the HyperScript™ RT Master Mix (GeneAll Biotechnology Co., Ltd., Seoul, Republic of Korea) and GeneAmp™ PCR System 9700 thermal cycler (Applied Biosystems, Carlsbad, CA, USA) as per the manufacturer’s recommendations.

After adding 5× HOT FIREpol^®^ EvaGreen^®^ qPCR Supermix (Solis BioDyne, Tartu, Estonia), qPCR was made using Applied Biosystems 7500 Real-Time PCR system (Thermo Fisher Scientific Inc., Waltham, MA, USA) with the following reaction conditions: 94 °C for 12 min followed by 40 cycles of 95 °C for 15 s, 65 °C for 30 s, and 72 °C for 30 s. GAPDH was used as an internal control to normalize the threshold cycle value and calculate the gene expression by calculating the 2^−ΔΔCT^ method [85]. Different independent RNA samples were used, and the experiment was repeated three times.

### 4.8. In Silico Molecular Docking Prediction of Combined Drugs

The chemical structure was obtained from the PubChem Compound database [86] for Res (PubChem ID: 445154) and Lov (PubChem ID: 53232). The ligand was optimized using Avogadro software (version 1.2.0) [87] and output to mol2 format. The crystal structures of interleukin-6 (IL-6) (PDB ID: 1ALU), transforming growth factor-β1 (TGF-β1) (PDB ID: 5VQP), and tumor necrosis factor-α (TNF-α) (PDB ID: 2AZ5) were obtained from the Protein Data Bank [88,89]. Protein preparation for docking was performed using AutoDockTools software (version 1.5.7) by water molecule and heteroatom elimination, repairing the missing atoms, and adding polar hydrogens and Kollman charges. The receptor coordinates, box sizes, and VINA parameters are outlined in Supplementary Table S1. The blind docking was performed using AutoDock Vina software (version 1.1.2) [90]. First, the best docking score and pose for the Lov receptor were determined; then, the output complex of the Lov receptor was prepared and docked with Res using the protocol mentioned above. Finally, the docking results of a complex Lov/Res-receptor were computed using the PyMol software (version 3.0.3). ChimeraX software (version 1.7.1) was used to generate 3D conformations, and the protein-ligand interactions analysis was performed using LigPlot software (version 2.2.8) [91].

### 4.9. Bacterial Strains and Culture

To examine the antibacterial potential of lovastatin and resveratrol on both Gram-negative *P. aeruginosa* prototroph strain PAO1, a contribution from Dr Lee Hughes from the University of North Texas, Texas, USA [75,92] and Gram-positive *S. aureus* (ATCC^®^ 29213) were used [93]. *S. aureus* cryovials were kindly provided by the Microbiology Laboratory, College of Dentistry Research Center, King Saud University, Riyadh, Saudi Arabia.

Bacterial stocks were taken from the −80 °C freezer and streaked onto a fresh nutrient agar plate for 24 h at 37 °C. Colony morphology was identified, and a single colony from each strain was obtained using a 10 µL loop and cultured in 10 mL nutrient broth (Oxoid, Hampshire, UK) using an Excella E24 incubator shaker (New Brunswick Scientific, Edison, NJ, USA) set at 100 rpm for 24 h at 37 °C. Using a spectrophotometer (Libra S22, Biochrom Ltd., Cambridge, UK), the bacterial count for both *S. aureus* and *P. aeruginosa* was standardized to OD600 = 0.5–0.6 (mid-log phase) [75].

### 4.10. Broth Microdilution Test for Bacterial Growth Inhibition

Following the Clinical and Laboratory Standards Institute (CLSI) protocol [94,95], different dilutions of lovastatin and resveratrol (0.05, 0.1, 0.25, 0.5, 1, 2, and 3 μM) were prepared in two separate rounded-bottom 96-well plates (Greiner Bio-One GmbH) for each drug concentration with six replicates.

Each well was filled with 150 μL nutrient broth containing the correct drug concentration. Then, 50 μL of the bacterial inoculate (OD600 = 0.5–0.6) was added to each well separately, resulting in four plates labeled as follows: Lov with *S. aureus*, Lov with *P. aeruginosa*, Res with *S. aureus*, and Res with *P. aeruginosa*. In addition, six wells of sterile nutrient broth and another six wells of 50 μL bacteria-containing nutrient broth were added to each plate as control wells.

After incubating the plates for 18 h at 37 °C, the Synergy™ HT Microplate Reader (BioTek^®^ Instruments, Winooski, VT, USA) was used to measure the absorbances at a wavelength of 600 nm [75,93].

The percentage of bacterial growth inhibition was calculated using the following formula after subtracting the value of OD600 from each reading:

(2) $$Bacterial\;growth\;inhibition\;\left(\%\right)=\;\frac{Average\;absorbance\;of\;the\;control\;wells-absorbance\;of\;the\;sample\;well}{Average\;absorbance\;of\;control\;wells}\;x\;100$$

### 4.11. Checkerboard Assay

Based on the CLSI protocol with some modifications [94,95,96,97], different dilutions of lovastatin (0.05, 0.1, 0.25, 0.5, 1, 2, and 3 μM) were placed vertically in two different U-shaped 96-well plates, whereas the diluted resveratrol solutions (0.05, 0.1, 0.25, 0.5, 1, 2, and 3 μM) were placed horizontally. The drugs were diluted to 150 μL nutrient broth per well. This was followed by adding 50 μL standardized bacterial inoculate (OD600 = 0.5–0.6) to each well. This method was performed independently for each strain used.

In addition, six wells were filled with sterile nutrient broth; another 12 wells were also filled with sterile nutrient broth mixed with trial drugs (6 wells/drug), and six wells of 50 μL of bacteria-containing broth and 150 μL of nutrient broth. The total volume for each well was 200 μL.

The plates were incubated at 37 °C for 18 h, and the optical densities at 600 nm were measured using the Synergy™ HT Microplate Reader (BioTek^®^ Instruments) [95,96]. The SynergyFinder Plus website package version 3.10.3 [44] calculated the synergy score and the half-maximal inhibitory concentration (IC50) for each drug.

### 4.12. Statistical Analysis

GraphPad Prism (Version 10.3.1, San Diego, CA, USA) was used to generate the graphs and analyze the data obtained from three independent experiments. Based on the data, one-way analysis of variance or two-way analysis of variance (ANOVA) was used to evaluate the significance, followed by either Dunnett’s post hoc test or Bonferroni’s post hoc analysis. The statistical significance was set as *p*-value <0.05.

## 5. Conclusions

Lovastatin at 0.1, resveratrol at 0.1 μM, and their combinations enhanced cell proliferation and wound-healing capability of the human TERT-20 mesenchymal stem cells and had the most minimal effect on their morphology. In addition, combining both lovastatin and resveratrol at a concentration of 0.1 μM had an additive effect against *S. aureus* and *P. aeruginosa*.

## Figures and Tables

**Table 1:** Summary of the combined ligands docking scores. The asterisk indicates the H-bond residue interaction of (*) Res and (**) Lov ligands.

Res/Lov-Receptor Complex	Affinity (kcal/mol^−1^)	Total H-Bonds	Residue—Length
IL-6	−6.0	2	ASP34—2.98 * GLU106—2.86 **
TGF-β1	−5.5	3	LYS77—2.77 * CYS264—2.95 ** CYS358—3.10 **
TNF-α	−6.5	4	TYR151—3.09 * LEU120—3.02 ** SER60—3.92 ** SER60—3.16 **

**Table 2:** The expression of wound-healing-related markers with their forward and reverse primers.

Target	Forward Primer Sequence	Reverse Primer Sequence
*IL-6*	5′-AGGAGACTTGCCTGGTGAAA-3′	5′-CAGGGGTGGTTATTGCATCT-3′
*TGF-β1*	5’-GGGACTATCCACCIGCAAGA-3′	5′-CCTCCTIGGCGTAGTAGICG-3′
*TNF-α*	5′-TCCTTCAGACACCCTCAACC-3′	5′-AGGCCCCAGTTTGAATTCTT-3′
*GAPDH*	5′-CAGCCTCCCGCTTCGCTCTC-3′	5′-CCAGGCGCCCAATACGACCA-3′

## Data Availability

All data generated from this research is presented in the publication. Any further inquiries can be directed to the corresponding author.

## Supplementary Materials

The following supporting information can be downloaded at: https://www.mdpi.com/article/10.3390/ijms26020851/s1.
