# Supplementary material for: Lovastatin and Resveratrol Synergistically Improve Wound Healing and Inhibit Bacterial Growth
Source: Int J Mol Sci. 2025 Jan 20;26(2):851. doi: 10.3390/ijms26020851 (PMC11766293; doi:10.3390/ijms26020851)
Supplement: Supplementary file 1 [file ijms-26-00851-s001.zip › ijms-3383288-supplementary.pdf]

Supplementary Table S1. Receptor coordinates and box sizes and AutoDock VINA parameters.

Ligand & receptor	Coordinates	Box size (Å)	Exhaustiveness	Energy range (kcal/mol)	Grid space
Lov-IL-6	x: 2.535<br>y: −19.890<br>z: 8.990	120 × 120 × 120	32	10	Default
Res/Lov-IL-6 complex					
Lov-TGF-β1	x: -13.360<br>y: 71.662<br>z: 27.035				
Res/Lov- TGF-β complex					
Lov- TNF-α	x: -13.360<br>y: 71.662<br>z: 27.035				
Res/Lov- TNF-α complex					

Supplementary Table S2. Molecular docking using VINA results. Asterisk indicates the residue interaction of (*) Res and (**) Lov ligands.

Res/Lov-receptor complex	Affinity (kcal/mol^{-1})	Total H-bonds	Residue – Length (Interaction)
IL-6	-6.0	2	ASP34 – 2.98 (H-Bond)*<br>ARG179 – (vdW)*<br>LEU178 – (vdW)*<br>GLN175– (vdW)*<br>LEU33 – (vdW)*<br>SER37 – (vdW)*<br><br>GLU106 – 2.86** (H-Bond)<br>LYS46 – (vdW)**<br>THR43 – (vdW)**<br>ARG104 – (vdW)**<br>GLUE42 – (vdW)**<br>PHE105 – (vdW)**<br>ASP160 – (vdW)**<br>GLN156 – (vdW)**
TGF-β1	-5.5	3	LYS77 – 2.77 (H-Bond) *<br>LEU242 – (vdW)*<br>ASP73 – (vdW)*<br>ALA239 – (vdW)*<br>LEU236 – (vdW)*<br>GLUE117 – (vdW)*<br>LEU120 – (vdW)*

			CYS264 – 2.95 (H-Bond) **<br>CYS358 – 3.10 (H-Bond) **<br>ASN263 – (vdW)**<br>PRO298 – (vdW)**<br>CYS360 – (vdW)**<br>LYS359 – (vdW)**<br>LEU313 – (vdW)**<br>PRO319 – (vdW)**<br>TYR74 – (vdW)**
TNF-α	-6.5	4	TYR151 – 3.09 (H-Bond)*<br>TYR59 – (vdW)*<br>LEU120 – (vdW)*<br>GLY121 – (vdW)*<br>SER60 – (vdW)*<br>TYR151 – (vdW)*<br>TYR119 – (vdW)*<br>TYR59 – (vdW)*<br>GLN61 – (vdW)*<br><br>LEU120 – 3.02 (H-Bond)**<br>SER60 – 3.92 (H-Bond)**<br>SER60 – 3.16 (H-Bond)**<br>SER60 – (vdW)**<br>LEU151 – (vdW)**<br>LEU120 – (vdW)**<br>GLN61 – (vdW)**<br>TYR119 – (vdW)**<br>LEU57 – (vdW)**<br>TYR119 – (vdW)**<br>GLY121 – (vdW)**<br>TYR121 – (vdW)**<br>TYR59 – (vdW)**

Supplementary Table S3. Protein-ligand molecular docking results.

Ligand-receptor complexes	Energy (kcal/mol^{-1})
Lov-IL6	-5.8
Res-IL6	-5.6
Res/Lov-IL6	-6.0
Lov- TGF-β1	-6.4
Res- TGF-β1	-5.9
Res/Lov- TGF-β1	5.5
Lov-TNF-α	-8.1
Res-TNF-α	-6.8
Res/Lov- TNF-α	-6.5
